# Fecal carriage of carbapenemase-producing *Escherichia coli* and *Klebsiella* spp. and associated factors among dairy farm milkers in Tigray Region, Northern Ethiopia

**DOI:** 10.1186/s12866-026-04943-7

**Published:** 2026-03-13

**Authors:** Goyitom Gebremedhn Gebru, Saravanan Muthupandian, Enquebaher Kassaye

**Affiliations:** 1 Department of Medical Microbiology, Tigray Health Research Institute, Mekelle, Tigray, 1547 Ethiopia; 2 Department of Veterinary Public Health and Food Safety, College of Veterinary Sciences, Mekelle University, Mekelle, Tigray, Ethiopia; 3 Department of Medical Laboratory Technology, Faculty of Applied Medical Sciences, University of Tabuk, Tabuk, 71491 Saudi Arabia; 4 Prince Fahad bin Sultan Chair for Biomedical Research, University of Tabuk, 71491 Tabuk , Saudi Arabia

**Keywords:** Carbapenemase, E. coli, *Klebsiella* spp., Dairy Farms, Fecal Carriage, Ethiopia

## Abstract

**Background:**

Antimicrobial resistance (AMR) among foodborne and zoonotic bacteria has become a major public health challenge. Dairy farm milkers can harbor resistant bacteria, potentially contributing to raw milk contamination and posing a risk to the wider community. This study aimed to assess the occurrence of fecal carriage and associated factors of carbapenemase-producing (CP) *Escherichia coli* (*E. coli*) and *Klebsiella* spp among dairy farm milkers in the Tigray region, Northern Ethiopia.

**Methods:**

A cross-sectional study was conducted from December 2024 to May 2025 among 178 dairy farm milkers. Stool samples were collected and analyzed for isolation and identification of *E. coli* and *Klebsiella* spp. using standard bacteriological methods. Antimicrobial susceptibility was determined using the Kirby–Bauer disk diffusion method. Carbapenemase production was confirmed phenotypically. Data on socio-demographics, hygiene practices, and food consumption were collected using a structured questionnaire. Firth’s penalized logistic regression was used to identify factors associated with fecal carriage of CP *E. coli* and *Klebsiella* spp.

**Results:**

*Escherichia coli* and *Klebsiella* spp were isolated from 121(68.0%) and 17(9.5%) of study participants. Carbapenemase-producing isolates were detected in 6 (3.4%) of the dairy farm milkers, all of which exhibited multidrug resistance. Multivariable analysis identified recent diarrhea (AOR = 23.02; 95% CI: 3.15–168.24) and raw milk consumption (AOR = 6.35; 95% CI: 1.07–37.46) as independent factors significantly associated with fecal carriage of CP *E. coli* and *Klebsiella* spp.

**Conclusion:**

Fecal carriage of CP *E. coli* and *Klebsiella* spp. was observed among dairy farm milkers. These findings highlight the potential food safety and public health relevance of antimicrobial-resistant bacteria carriage in dairy farm workers and support the inclusion of milkers in One Health-oriented AMR surveillance and hygiene-focused interventions along the dairy value chain.

**Supplementary Information:**

The online version contains supplementary material available at 10.1186/s12866-026-04943-7.

## Background

Antimicrobial resistance among foodborne and zoonotic bacteria is an increasing threat to public health and food safety worldwide [1]. In particular, carbapenemase-producing Enterobacterales, especially *Escherichia coli* (*E. coli*) and *Klebsiella* spp have become a major concern. These bacteria gained global attention because they can inactivate critically important antimicrobials and circulate across human, animal, food, and environmental settings [2, 3]. Intestinal colonization of humans by these resistant bacteria is considered an important factor in their persistence within communities and may facilitate their dissemination [4, 5].

This challenge becomes more complex within food production systems. Dairy farms represent a key point of interaction between humans, animals, and food products, creating favorable conditions for zoonotic exchange of antimicrobial-resistant bacteria [6–8]. Milkers and dairy farm workers are routinely exposed to animal feces, contaminated surfaces, water, and raw milk during daily activities [9, 10]. Fecal carriage of antimicrobial-resistant *E. coli* and *Klebsiella* spp among milkers is therefore of particular concern, as it may increase the risk of contamination of milk during handling [11, 12]. This suggests a possible pathway between human intestinal colonization and foodborne exposure in consumers, highlighting the role of farm workers as critical nodes in the transmission chain of antimicrobial resistance within the dairy value chain [13, 14].

These risks are amplified in settings with limited sanitation and inconsistent hygiene practices. In many low-resource areas, poor hand hygiene and the frequent consumption of raw foods contribute to the persistent community carriage of resistant bacteria [15, 16]. Several studies from Africa have reported increasing fecal carriage of carbapenemase-producing *E. coli* and *Klebsiella* spp among food handlers and agricultural workers [17, 18]. Despite this, human carriage of antimicrobial-resistant bacteria has received limited attention in food safety surveillance systems, which traditionally focus on animal or food samples while overlooking the role of food handlers.

In Ethiopia, dairy production continues to expand while raw milk consumption remains common [19, 20]. Previous studies in the country have reported antimicrobial resistance, including resistance to third-generation cephalosporins and carbapenems, in *E. coli* and *Klebsiella* spp. from milk, dairy farm environments, and animal feces [21–23]. However, the role of milkers as reservoirs and sources of resistant *E. coli* and *Klebsiella* spp has received limited attention, particularly in the Tigray region, northern Ethiopia. Additionally, there are no existing studies that focus on the carriage of carbapenemase-producing *E. coli* and *Klebsiella* spp among milkers in Ethiopia, particularly in Tigray. This knowledge gap limits the ability to design effective interventions targeting human-mediated contamination along the dairy value chain.

In the Tigray region, where dairy farms supply raw milk to a large urban population, understanding fecal carriage of CP *E. coli* and *Klebsiella* spp among milkers is essential from a One Health perspective. Therefore, this study aimed to determine the prevalence of fecal carriage of CP *E. coli* and *Klebsiella* spp among dairy farm milkers in Tigray, Northern Ethiopia, and to identify associated factors with direct implications for milk safety and zoonotic transmission.

## Materials and methods

### Study design and setting

**Fig. 1:**
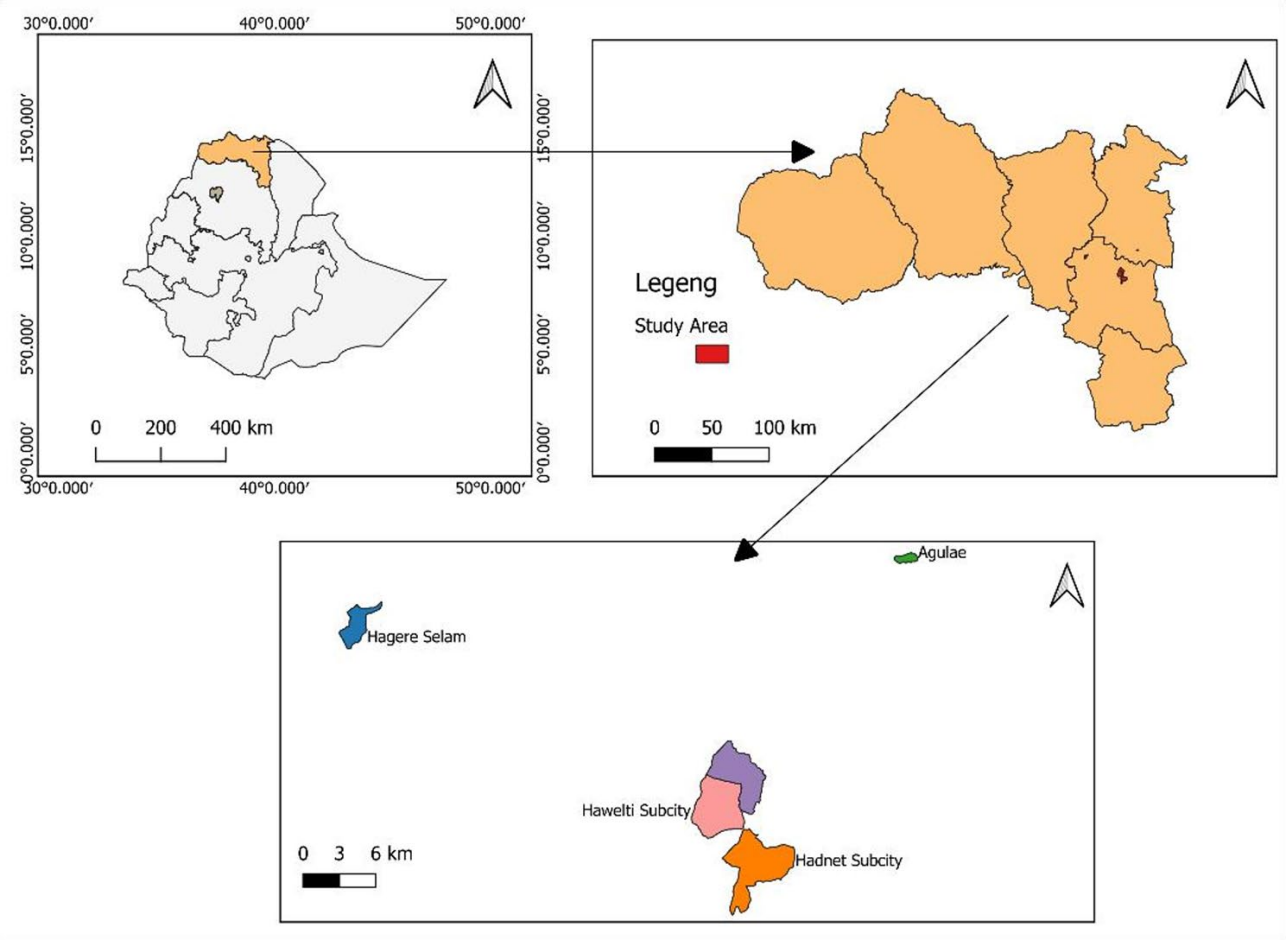
Map of the study area in Tigray Region, Northern Ethiopia

This current study is part of a larger study [24], conducted on dairy farms to investigate antimicrobial resistance of *E. coli* and *Klebsiella* spp in a One Health approach. Briefly, a cross-sectional study was carried out from December 2024 to May 2025 among dairy farm milkers in selected areas of the Tigray region, Northern Ethiopia. The study was conducted in three administrative zones, Eastern Zone, the Southeastern Zone, and the Mekelle Special Zone. Five woredas or sub-cities were selected, including Ayder, Hadnet, and Hawelti from Mekelle, Agulae from the Eastern Zone, and Hagereselam from the Southeastern Zone, as shown in Fig. 1.

Mekelle city, Agulae, and Hagereselam were purposively selected as study sites to represent the segments of the regional milk value chain. Mekelle was included for its status as the main urban center, characterized by high milk consumption, a dense concentration of dairy farms, and a significant volume of raw milk supply. Agulae and Hagereselam were selected as they are major rural milk production areas that supply raw milk to Mekelle city. In these areas, dairy farming is primarily smallholder-based, with close interaction between humans and animals.

### Sample size and sampling technique

This study used the human stool component of a larger dairy farm investigation conducted in the same study areas [24]. The sample size was determined as 178 dairy farm milkers. One milker from each dairy farm who was actively involved in milking, milk handling, or farm hygiene activities at the time of the survey and who consented to participate was included.

### Stool sample collection

Fresh stool samples were collected from dairy farm milkers using clean, wide-mouthed, sterile stool containers. Each container was labeled with a unique identification code of the participant and the date of collection. The stool samples were placed in Cary-Blair transport medium and transported in insulated cool boxes with ice packs to the Microbiology Laboratory of the Tigray Health Research Institute within four hours for bacteriological analysis.

### Isolation and identification of *E. coli* and *Klebsiella* spp

Isolation and identification of *E. coli* and *Klebsiella* spp were performed using standard bacteriological methods in accordance with established laboratory guidelines. Briefly, stool was streaked onto MacConkey agar and incubated at 37 °C for 24–48 h. Presumptive lactose-fermenting colonies were selected based on colony morphology and Gram staining results. Pure cultures were obtained by subculturing on Tryptic Soy Agar.

A panel of biochemical tests, including Triple Sugar Iron reaction, indole production, citrate utilization, urease activity, and motility, was used to differentiate *E. coli* and *Klebsiella* species.

## Antimicrobial susceptibility testing

Antimicrobial susceptibility testing was conducted using the Kirby–Bauer disk diffusion method following Clinical and Laboratory Standards Institute guidelines [25]. Pure bacterial colonies were suspended in sterile normal saline and adjusted to match a 0.5 McFarland turbidity standard. The suspension was inoculated onto Mueller–Hinton agar using the lawn culture technique.

The following antimicrobial agents were tested: Trimethoprim/Sulphamethoxazole (SXT, 23.75 µg /1.25 µg), Ciprofloxacin (CIP, 5 µg), Tetracycline (TE, 30 µg), Piperacillin/Tazobactam (TZP, 110 µg), Amoxicillin/clavulanic acid (AMC, 20/10 µg), Ampicillin/Sulbactam (SAM, 30 µg), Cefotaxime (CTX, 30 µg), Ceftazidime (CAZ, 30 µg), Ceftriaxone (CRO, 30 µg), Cefoxitin (FOX, 30 µg), Aztreonam (ATM, 30 µg), Amikacin (AK, 30 µg), and Meropenem (MEM, 10 µg). The plates were incubated at 37 °C for 16–18 h, after which inhibition zone diameters were measured and interpreted using CLSI 2024 [25]. Multidrug resistance (MDR) was defined as resistance to one or more antimicrobial agents in three or more different antimicrobial classes [26].

### Detection and confirmation of carbapenemase production

Screening for carbapenemase production was performed among *E. coli* and *Klebsiella* spp. isolates using phenotypic antimicrobial susceptibility testing. Isolates showing reduced susceptibility (≤ 19 mm for meropenem) were considered suspected carbapenemase producers, in accordance with Clinical and Laboratory Standards Institute (CLSI) guidelines [25].

*Escherichia coli* and *Klebsiella* spp isolates meeting the screening criteria were subjected to confirmatory testing using the modified carbapenem inactivation method (mCIM) [25]. Briefly, a 1 µL loopful of the test isolate was emulsified in 2 mL of Tryptic Soy Broth (TSB), followed by the immersion of a 10 µg meropenem disk. After a 4-hour incubation at 35 °C, the disk was removed and placed onto a Mueller-Hinton Agar (MHA) plate previously inoculated with a 0.5 McFarland suspension of the susceptible indicator strain, *Escherichia coli* ATCC 25,922. A zone of inhibition measuring between 6 and 15 mm after 18–24 h of incubation was interpreted as positive for carbapenemase production.

### Quality assurance

Quality control procedures were applied throughout the laboratory process. Reference strains *E. coli* ATCC 25,922 and *K. pneumoniae* ATCC 700,603 were used to verify the performance of culture media, biochemical tests, and antimicrobial susceptibility testing. All laboratory procedures were conducted by trained personnel following standard operating procedures.

### Questionnaire survey and risk factor assessment

A structured questionnaire was administered to dairy farm milkers to collect data on potential factors associated with fecal carriage of carbapenemase-producing bacteria (**Additional file 1**). Data were collected using Open Data Kit on Android devices and verified through direct observation where appropriate.

The questionnaire captured information on socio-demographic characteristics, personal hygiene practices, sanitation facilities, food consumption behaviors, health history, and occupational exposure related to dairy farming activities.

### Data processing and analysis

Laboratory and questionnaire data were entered into Microsoft Excel and analyzed using Stata version 16. Descriptive statistics were used to summarize participant characteristics and the prevalence of CP *E. coli* and *Klebsiella* spp.

To identify factors associated with fecal carriage of CP *E. coli* and *Klebsiella* spp, Firth’s penalized logistic regression was used due to the low prevalence of the outcome. To minimize overfitting, the number of predictors was restricted, and variables were selected a priori based on biological plausibility and evidence from previous studies, which include recent hospital admission, recent diarrhea or UTI, hygiene practices (fingernail trimming), dietary behavior (raw milk consumption), and knowledge of diseases transmitted by raw milk. Socio-demographic variables and other environmental factors were described descriptively but were not included in the multivariable model. Results were reported as adjusted odds ratios (AORs) with 95% confidence intervals, and statistical significance was assessed at *p* *≤* 0.05.

## Result

A total of 178 dairy farm milkers were included in this study. The majority were male 147(82.6%) and aged ≤ 35 years 107(60.1%). Approximately two-thirds of participants had a low level of formal education 119(66.9%) (Table 1).

**Table 1:** Socio-demographic, clinical, and hygienic characteristics of milkers in dairy farms in the Tigray region, Northern Ethiopia, (December 2024 to May 2025)

Variable	Category	Frequency (*n* = 178)	Percent (%)
Sex	Male	147	82.6
	Female	31	17.4
Age in years	<=35	107	60.1
	> 35	71	39.9
Education level	Low Education level	119	66.9
	High Education Level	59	33.1
Regular health check-up	No	162	91.0
	Yes	16	9.0
History of medical instrumentation	Yes	3	1.7
	No	175	98.3
Hospital admission in the last 3 months	Yes	12	6.7
	No	166	93.3
Diarrhea in the last 3 months	Yes	16	9.0
	No	162	91.0
Urinary tract infection in the last 3 months	Yes	11	6.2
	No	167	93.8
Availability of hand-washing facility	No	151	84.8
	Yes	27	15.2
Hand washing before meals and after toilet use	No	143	80.3
	Yes	35	19.7
Fingernail trimmed	No	10	5.6
	Yes	168	94.4
Source of drinking water	Unimproved	110	61.8
	Improved	68	38.2
Availability of latrine facility	No	117	65.7
	Yes	61	34.3
Raw milk consumption	Yes	39	21.9
	No	139	78.1
Raw meat consumption	Yes	6	3.4
	No	172	96.6
Raw vegetable consumption	Yes	25	14.1
	No	153	85.9
Knowledge of diseases transmitted by raw milk	No	109	61.2
	Yes	69	38.8

The dairy farm milkers had limited access to safe water and sanitation, with 110(61.8%) of milkers relying on unimproved sources of drinking water and 117(65.7%) lacking access to latrine facilities. In addition, the majority of participants, 148(83.1%), reported not consistently washing their hands before meals and after using the toilet (Table 1**).**

Among the participants, raw milk consumption was reported by 39(21.9%) of milkers, and no knowledge of diseases transmitted through raw milk was reported by 109(61.2%) (Table 1).

### Prevalence of carbapenemase-producing *E. coli* and *Klebsiella* spp

Among the 178 stool samples collected from dairy farm milkers, CP isolates were found in only 6 samples, representing 3.4%. Among these, 5 (83.3%) were *E. coli* and 1 (16.7%) was *Klebsiella* spp.

### Antimicrobial resistance patterns of carbapenemase-producing *E. coli* and *Klebsiella* spp

Among the CP isolates (*N* = 6), high levels of co-resistance to β-lactam antibiotics were observed. Resistance to amoxicillin–clavulanic acid and cefotaxime was 100%, while slightly lower resistance was observed for ceftazidime and ceftriaxone, 83.3% each. Resistance to non-β-lactam antimicrobials was variable, with 83.3% for tetracycline, and 66.6% each for ciprofloxacin and trimethoprim–sulfamethoxazole, and lower resistance to amikacin (33.3%) (Table 2).

**Table 2:** Antimicrobial resistance profiles of carbapenemase-producing *Escherichia coli* and *Klebsiella* spp. isolated from stool samples of dairy farm milkers in the Tigray region, Northern Ethiopia, (December 2024 to May 2025)

Bacterial isolates	carbapenemase-producing (*N* = 6)	AMC *R* (*N*(%))	TZP *R* (*N*(%))	SAM *R* (*N*(%))	CTX *R* (*N*(%))	CAZ *R* (*N*(%))	CRO *R* (*N*(%))	FOX *R* (*N*(%))	ATM *R* (*N*(%))	AK *R* (*N*(%))	MEM *R* (*N*(%))	TE *R* (*N*(%))	CIP *R* (*N*(%))	SXT *R* (*N*(%))
*E. coli*	**5**	5(100)	4(80)	4(80)	5(100)	4(80)	4(80)	4(80)	4(80)	1(20)	5(100)	5(100)	3(60)	3(60)
*Klebsiella spp.*	**1**	1(100)	0(0.0)	0(0.0)	1(100)	1(100)	1(100)	1(100)	1(100)	1(100)	1(100)	0(0.0)	1(100)	1(100)
Total	**6**	6(100)	4(66.6)	4(66.6)	6(100)	5(83.3)	5(83.3)	5(83.3)	5(83.3)	2(33.3)	6(100)	5(83.3)	4(66.6)	4(66.6)

Note: Percentages are calculated within species; percentages in the total row are calculated using the overall number of carbapenemase-producing isolates (*N* = 6).

Key: *R *Resistant, *AMC *Amoxicillin/clavulanic acid, *TZP *Piperacillin/Tazobactam, *SAM *Ampicillin/Sulbactam, *CTX *Cefotaxime, *CAZ *Ceftazidime, *CRO *Ceftriaxone, *FOX *Cefoxitin, *ATM *Aztreonam, *AK *Amikacin, *MEM *Meropenem, *TE *Tetracycline, *CIP *Ciprofloxacin, *SXT *Trimethoprim/Sulphamethoxazole

### Factors associated with fecal carriage of carbapenemase-producing *E. coli* and *Klebsiella* Spp

In this study, factors associated with fecal carriage of CP *E. coli* and *Klebsiella* spp were assessed among 178 dairy farm milkers, as indicated in Table 1. The variables assessed included socio-demographic characteristics, personal hygiene, health history, water and sanitation facilities, and food consumption practices. Due to the low prevalence of the outcome, Firth’s penalized logistic regression was used to provide stable estimates. Therefore, from the assessed variables, only variables with biological plausibility and support from previous literature, including hospital admission in the last 3 months, diarrhea in the last 3 months, urinary tract infection in the last 3 months, fingernail status, and raw milk consumption, were included in the multivariable model to determine factors independently associated with fecal carriage of CP organisms (Table 3).

**Table 3:** Multivariable Firth’s penalized logistic regression analysis of factors associated with fecal carriage of carbapenemase-producing *E. coli* and *Klebsiella* spp among dairy farm milkers in the Tigray region, Northern Ethiopia (December 2024–May 2025)

Variable	Category	Carbapenemase-positive, *n* (%)	AOR (95% CI)	*p*-value
Hospital admission in the last 3 months	Yes	1(8.3)	9.94(0.98–100.80)	0.052
	No	5(3.0)	1.00	Reference
Diarrhea in the last 3 months	Yes	3(18.8)	23.02(3.15-168.24)	0.002^*^
	No	3(1.9)	1.00	Reference
Urinary tract infection in the last 3 months	Yes	1(9.1)	0.74(0.05–9.97)	*0.821*
	No	5(3.0)	1.00	Reference
Fingernail trimmed	No	1(10.0)	4.15(0.40-42.95)	0.232
	Yes	5(3.0)	1.00	Reference
Raw milk consumption	Yes	3(7.7)	6.35(1.07–37.46)	0.041^*^
	No	3(2.2)	1.00	Reference
Knowledge of diseases transmitted by raw milk	No	2(1.8)	0.27(0.04–1.70)	0.166
	Yes	4(5.8)	1.00	Reference

*AOR*→ Adjusted Odds Ratio, *CI*→ Confidence Interval, ^*^ → significantly associated

In the multivariable Firth’s penalized logistic regression analysis, a history of diarrhea in the past three months and raw milk consumption were independently associated with fecal carriage of CP *E. coli* and *Klebsiella spp*. Milkers who reported recent diarrhea had higher odds of carriage compared to those without such a history (AOR = 23.02; 95% CI: 3.15–168.24). Similarly, those who consumed raw milk had significantly higher odds of carriage (AOR = 6.35; 95% CI: 1.07–37.46) (Table 3).

Participants with a history of hospital admission in the past three months had higher odds of fecal carriage compared to those without hospital admission (AOR = 9.94; 95% CI: 0.98–100.80), although this was not statistically significant. Urinary tract infection within the last three months, fingernail status, and knowledge of diseases transmitted by raw milk were not significantly associated with fecal carriage in the adjusted model (Table 3).

## Discussion

In this study, fecal carriage of carbapenemase-producing *E. coli* and *Klebsiella* spp. was observed among dairy farm milkers in the Tigray region, Ethiopia. Although the overall prevalence was relatively low (3.4%), the presence of these last-resort antibiotic-resistant organisms among dairy farm milkers is a significant public health concern. Dairy farm milkers operate at the interface of humans, animals, and food, yet they remain underrepresented in antimicrobial resistance surveillance. Therefore, this study provides human-centered evidence for AMR monitoring within the dairy value chain and highlights its implications for food safety.

Carbapenemase-producing isolates were detected at low frequency, consistent with community-based studies in Ethiopia and other African countries [17, 18]. The low prevalence likely reflects limited access to carbapenems in both the human and veterinary sectors, thereby reducing selection pressure [26–28]. However, even this low occurrence is significant because carbapenems are considered last-resort antibiotics for the treatment of severe infections [28].

Dairy farm milkers’ close and frequent contact with animals and raw milk may facilitate the spread and persistence of antimicrobial-resistant bacteria [29]. During milking and milk handling, resistant organisms can contaminate the milk through direct contact, unwashed hands, farm equipment, or the farm environment [30–32]. Although our study did not investigate genomic relatedness between human and milk isolates, these findings suggest the possibility of contamination pathways through which resistant bacteria could be transferred between milkers and raw milk, posing a potential risk of exposure to consumers.

In this study, among carbapenemase-producing isolates, high levels of co-resistance to β-lactam antibiotics, including 100% resistance to amoxicillin–clavulanic acid and cefotaxime, were observed. This pattern is common, as carbapenemase enzymes often coexist with other β-lactam resistance mechanisms, including extended-spectrum β-lactamases and AmpC β-lactamases [33, 34]. Resistance to commonly used non-β-lactam antibiotics such as tetracycline, ciprofloxacin, and trimethoprim-sulfamethoxazole was also substantial, suggesting multidrug resistance. However, relatively lower resistance to amikacin was observed, indicating that it may remain a potential therapeutic option.

In the multivariable analysis, a history of diarrhea in the past three months was found independently associated with fecal carriage of CP *E. coli* and *Klebsiella* spp. Individuals who reported recent diarrhea had significantly higher odds of carriage. This association may be due to several factors, including disruption of the normal gut microbiota, increased exposure to healthcare settings, or prior antimicrobial use during diarrheal episodes. These factors could facilitate the colonization or selection of resistant strains [35, 36]. Additionally, raw milk consumption was independently associated with fecal carriage. Consumption of unpasteurized milk may serve as a potential route of exposure to antimicrobial-resistant bacteria originating from dairy animals or contaminated environments [37–40]. In this study, a significant proportion of milkers reported consuming raw milk, and knowledge about diseases transmitted through raw milk was limited, consistent with other studies from Ethiopia and other low- and middle-income countries [41–43].

Similarly, dairy farming in Tigray is mostly small-scale and traditional, with limited access to veterinary services, inadequate sanitation, and minimal awareness of milk-borne diseases [44]. Milk is commonly consumed unpasteurized at both household and market levels [45]. These risky behaviors in the region where this study was conducted increase the risk of AMR transmission from dairy products to humans. Moreover, the limited availability of microbiology laboratories and surveillance systems in the Tigray region may create conducive settings for resistant bacteria to circulate undetected.

These findings contribute to a broader One Health perspective on antimicrobial resistance in Ethiopia and other similar low- and middle-income settings. Regional studies have provided evidence of multidrug-resistant, extended-spectrum β-lactamase, and CP-producing Enterobacterales found in livestock, raw milk, poultry, and environmental sources like water and soil [46–50]. Therefore, within this framework, the presence of carbapenemase-producing *E. coli* and *Klebsiella* spp. in the feces of dairy farm milkers in this study may indicate larger ecological trends at the intersection of human, animal, and environmental health in the dairy farm and beyond.

This study provides valuable baseline data contributing to a significant knowledge gap regarding human-focused dairy farms. However, this study has some limitations. First, the cross-sectional design restricts our ability to make causal inferences between risk factors and the presence of bacterial carriage. Additionally, we were unable to follow up with carriers to assess the persistence of colonization or any progression to infection. Furthermore, the investigation focused solely on the phenotypic detection of carbapenemase production in *E. coli* and *Klebsiella* spp., which likely resulted in an underestimation of the true prevalence. Finally, due to limited laboratory capacity, we did not perform molecular characterization of antimicrobial resistance genes.

## Conclusion

This study found fecal carriage of carbapenemase-producing *E. coli* and *Klebsiella* spp. among dairy farm milkers in the Tigray region, Ethiopia, with all isolates exhibiting multidrug resistance. Fecal carriage was strongly associated with recent diarrhea and raw milk consumption. This suggests the influence of gastrointestinal disturbances, antimicrobial exposure, and foodborne transmission pathways in maintaining and spreading resistant bacteria.

Dairy farm milkers may act as a source of contamination for raw milk and contribute to the dissemination of resistant bacteria within the community. Therefore, the findings from this study emphasize the importance of including milkers in One Health-oriented AMR surveillance and highlight the need for context-based interventions, including improved hygiene practices, promotion of safe milk consumption, and increased awareness of antimicrobial resistance and milk-borne diseases, particularly in low-resource settings like Tigray.

## Supplementary Information

Additional file 1: Questionnaire

## Data Availability

The datasets used and/or analyzed during the current study are available from the corresponding author on reasonable request.

## Abbreviations

AMR: Antimicrobial Resistance
AOR: Adjusted Odds Ratio
ATCC: American Type Culture Collection
CI: Confidence Interval
CLSI: Clinical and Laboratory Standards Institute
E. coli: Escherichia coli
MDR: Multidrug-Resistant
MHA: Muller-Hinton Agar
ODK: Open Data Kit
OR: Odds Ratio
THRI: Tigray Health Research Institute

## References

[1] Elbehiry A, Marzouk E, Abalkhail A, Edrees HM, Ellethy AT, Almuzaini AM, et al. Microbial Food Safety and Antimicrobial Resistance in Foods: A Dual Threat to Public Health. Microorganisms. 2025;13(7):1592. 10.3390/microorganisms13071592. 40732101 10.3390/microorganisms13071592 PMC12299635

[2] Aljohni MS, Harun-Ur-Rashid M, Selim S. Emerging threats: Antimicrobial resistance in extended-spectrum beta-lactamase and carbapenem-resistant Escherichia coli. Microb Pathog. 2025;200:107275. 10.1016/j.micpath.2024.107275. 39798725 10.1016/j.micpath.2024.107275

[3] Ramatla T, Mafokwane T, Lekota K, Monyama M, Khasapane G, Serage N, et al. One Health perspective on prevalence of co-existing extended-spectrum β-lactamase (ESBL)-producing Escherichia coli and Klebsiella pneumoniae: a comprehensive systematic review and meta-analysis. Ann Clin Microbiol Antimicrob. 2023;22(1):88. 10.1186/s12941-023-00638-3. 37740207 10.1186/s12941-023-00638-3 PMC10517531

[4] Abera D, Negash AA, Fentaw S, Mekonnen Y, Cataldo RJ, Wami AA, et al. High prevalence of colonization with extended-spectrum beta-lactamase-producing and multidrug-resistant Enterobacterales in the community in Addis Ababa Ethiopia: risk factors, carbapenem resistance, and molecular characterization. BMC Microbiol. 2024;24(1):402. 10.1186/s12866-024-03552-6. 39390409 10.1186/s12866-024-03552-6 PMC11465526

[5] Hu Y, Matsui Y, L WR. Risk factors for fecal carriage of drug-resistant Escherichia coli: a systematic review and meta-analysis. Antimicrob Resist Infect Control. 2020;9(1):31. 10.1186/s13756-020-0691-3. 32046786 10.1186/s13756-020-0691-3 PMC7014593

[6] Trinchera M, De Gaetano S, Sole E, Midiri A, Silvestro S, Mancuso G, et al. Antimicrobials in Livestock Farming and Resistance: Public Health Implications. Antibiot (Basel). 2025;14(6):606. 10.3390/antibiotics14060606. 10.3390/antibiotics14060606 PMC12190098 40558196

[7] Olaru ID, Walther B, Schaumburg F. Zoonotic sources and the spread of antimicrobial resistance from the perspective of low and middle-income countries. Infect Dis Poverty. 2023;12(1):59. 10.1186/s40249-023-01113-z. 37316938 10.1186/s40249-023-01113-z PMC10265791

[8] Magnusson U, Moodley A, Osbjer K. Antimicrobial resistance at the livestock-human interface: implications for Veterinary Services. Rev Sci Tech. 2021;40(2):511–21. 10.20506/rst.40.2.3241. 34542097 10.20506/rst.40.2.3241

[9] Linke L, Magnuson R, McConnel C, Palomares Velosa J, Rao S, Reynolds S, et al. Socio-Ecological Factors of Zoonotic Diseases Exposure in Colorado Dairy Workers. J Agromedicine. 2021;26(2):151–61. 10.1080/1059924x.2020.1725700. 32052708 10.1080/1059924X.2020.1725700 PMC9552966

[10] Holzhauer M, Wennink GJ. Zoonotic risks of pathogens from dairy cattle and their milk-borne transmission. J Dairy Res. 2023;90(4):325–31. 10.1017/s0022029923000730. 38186208 10.1017/S0022029923000730

[11] Dahms C, Hubner NO, Kossow A, Mellmann A, Dittmann K, Kramer A. Occurrence of ESBL-Producing Escherichia coli in Livestock and Farm Workers in Mecklenburg-Western Pomerania, Germany. PLoS ONE. 2015;10(11):e0143326. 10.1371/journal.pone.0143326. 26606146 10.1371/journal.pone.0143326 PMC4659621

[12] Diriba K, Awulachew E, Tekele L, Ashuro Z. Fecal carriage rate of extended-spectrum beta-lactamase-producing Escherichia coli and Klebsiella pneumoniae among apparently health food handlers in Dilla University student cafeteria. Infect Drug Resist. 2020;13:3791–800. 10.2147/idr.s269425. 33122924 10.2147/IDR.S269425 PMC7590998

[13] Muloi D, Ward MJ, Pedersen AB, Fevre EM, Woolhouse ME, van Bunnik BA. Are food animals responsible for transfer of antimicrobial-resistant Escherichia coli or their resistance determinants to human populations? A systematic review. Foodborne Pathog Dis. 2018;15(8):467–74. 10.1089/fpd.2017.2411. 29708778 10.1089/fpd.2017.2411 PMC6103250

[14] Elbehiry A, Marzouk E. From Farm to Fork: Antimicrobial-Resistant Bacterial Pathogens in Livestock Production and the Food Chain. Vet Sci. 2025;12(9):862. 10.3390/vetsci12090862. 41012787 10.3390/vetsci12090862 PMC12474250

[15] Ramay BM, Caudell MA, Cordon-Rosales C, Archila LD, Palmer GH, Jarquin C, et al. Antibiotic use and hygiene interact to influence the distribution of antimicrobial-resistant bacteria in low-income communities in Guatemala. Sci Rep. 2020;10(1):13767. 10.1038/s41598-020-70741-4. 32792543 10.1038/s41598-020-70741-4 PMC7426860

[16] Vicar EK, Alo DB, Koyiri VC, Opare-Asamoah K, Obeng-Bempong M, Mensah GI. Carriage of antibiotic resistant bacteria and associated factors among food handlers in Tamale Metropolis, Ghana: Implications for food safety. Microbiol Insights. 2023;16:11786361221150695. 10.1177/11786361221150695. 36726578 10.1177/11786361221150695 PMC9885032

[17] Amare A, Eshetie S, Kasew D, Moges F. High prevalence of fecal carriage of Extended-spectrum beta-lactamase and carbapenemase-producing Enterobacteriaceae among food handlers at the University of Gondar, Northwest Ethiopia. PLoS ONE. 2022;17(3):e0264818. 10.1371/journal.pone.0264818. 35298493 10.1371/journal.pone.0264818 PMC8929611

[18] Sanneh B, Kebbeh A, Jallow HS, Camara Y, Mwamakamba LW, Ceesay IF, et al. Prevalence and risk factors for faecal carriage of Extended Spectrum β-lactamase producing Enterobacteriaceae among food handlers in lower basic schools in West Coast Region of The Gambia. PLoS ONE. 2018;13(8):e0200894. 10.1371/journal.pone.0200894. 30102698 10.1371/journal.pone.0200894 PMC6089431

[19] Deneke TT, Bekele A, Moore HL, Mamo T, Almaw G, Mekonnen GA, et al. Milk and meat consumption patterns and the potential risk of zoonotic disease transmission among urban and peri-urban dairy farmers in Ethiopia. BMC Public Health. 2022;22(1):222. 10.1186/s12889-022-12665-4. 35114957 10.1186/s12889-022-12665-4 PMC8815239

[20] Duguma B. Milk composition, traditional processing, marketing, and consumption among smallholder dairy farmers in selected towns of Jimma Zone, Oromia Regional State, Ethiopia. Food Sci Nutr. 2022;10(9):2879–95. 10.1002/fsn3.2884. 36171770 10.1002/fsn3.2884 PMC9469845

[21] Beyene AM, Gizachew M, Yousef AE, Haileyesus H, Abdelhamid AG, Berju A, et al. Multidrug-resistance and extended-spectrum beta-lactamase-producing lactose-fermenting enterobacteriaceae in the human-dairy interface in northwest Ethiopia. PLoS ONE. 2024;19(5):e0303872. 10.1371/journal.pone.0303872. 38771780 10.1371/journal.pone.0303872 PMC11108214

[22] Torka TT. Extended-Spectrum Beta-Lactamase Producing Escherichia coli in Raw Cow Milk At Selling Points and Determinants of Contamination in and Around Chencha, Southern Ethiopia. Vet Med (Auckl). 2024;15:159–69. 10.2147/vmrr.s454930. 38784220 10.2147/VMRR.S454930 PMC11112127

[23] Muhummed A, Alemu A, Hosch S, Osman Y, Tschopp R, Yersin S, et al. Fecal carriage of ESBL-producing E. coli and genetic characterization in rural children and livestock in the Somali region, Ethiopia: a one health approach. Antimicrob Resist Infect Control. 2024;13(1):148. 10.1186/s13756-024-01502-5. 39695886 10.1186/s13756-024-01502-5 PMC11656975

[24] Gebru GG, Muthupandian S, Kassaye E. Prevalence and antimicrobial resistance of Escherichia coli and Klebsiella spp in dairy farm in the Tigray Region, Northern Ethiopia. BMC Microbiol. 2025;25(1):12. 10.1186/s12866-025-04643-8. 41422193 10.1186/s12866-025-04643-8 PMC12836893

[25] CLSI. Performance standards for Antimicrobial Susceptibility Testing. 34th ed. CLSI supplement M100. Clinical and Laboratory Standards Institute; 2024.

[26] Huang E, Yang X, Leighton E, Li X. Carbapenem resistance in the food supply chain. J Food Prot. 2023;86(7):100108. 10.1016/j.jfp.2023.100108. 37244353 10.1016/j.jfp.2023.100108

[27] Ramírez-Castillo FY, Guerrero-Barrera AL, Avelar-González FJ. An overview of carbapenem-resistant organisms from food-producing animals, seafood, aquaculture, companion animals, and wildlife. Front Vet Sci. 2023;10:1158588. 10.3389/fvets.2023.1158588. 37397005 10.3389/fvets.2023.1158588 PMC10311504

[28] Alvisi G, Curtoni A, Fonnesu R, Piazza A, Signoretto C, Piccinini G, et al. Epidemiology and genetic traits of carbapenemase-producing enterobacterales: a global threat to human health. Antibiot (Basel). 2025;14(2):141. 10.3390/antibiotics14020141. 10.3390/antibiotics14020141 PMC11852015 40001385

[29] Elbehiry A, Marzouk E. From farm to fork: antimicrobial-resistant bacterial pathogens in livestock production and the food chain. Vet Sci. 2025;12(9):862. 10.3390/vetsci12090862. 41012787 10.3390/vetsci12090862 PMC12474250

[30] Owusu-Kwarteng J, Akabanda F, Agyei D, Jespersen L. Microbial safety of milk production and fermented dairy products in Africa. Microorganisms. 2020;8(5):752. 10.3390/microorganisms8050752. 32429521 10.3390/microorganisms8050752 PMC7285323

[31] Deddefo A, Mamo G, Leta S, Amenu K. Prevalence and molecular characteristics of Staphylococcus aureus in raw milk and milk products in Ethiopia: a systematic review and meta-analysis. Int J Food Contam. 2022;9(1):8. 10.1186/s40550-022-00094-5.

[32] Sarba EJ, Wirtu W, Gebremedhin EZ, Borena BM, Marami LM. Occurrence and antimicrobial susceptibility patterns of Escherichia coli and Escherichia coli O157 isolated from cow milk and milk products. Ethiopia Sci Rep. 2023;13:16018. 10.1038/s41598-023-43043-8. 37749163 10.1038/s41598-023-43043-8 PMC10519974

[33] Garba Z, Bonkoungou IJ, Somda NS, Natama MH, Somé G, Sangaré L, et al. Fecal carriage of carbapenemase and AmpC-β-lactamase producers among extended spectrum β-lactamase-producing E. coli and Klebsiella spp. isolates in patients attending hospitals. BMC Infect Dis. 2025;25:109. 10.1186/s12879-025-10506-4. 39849346 10.1186/s12879-025-10506-4 PMC11760080

[34] Garba Z, Kaboré B, Bonkoungou IJ, Natama MH, Rouamba T, Haukka K, et al. Phenotypic detection of carbapenemase and AmpC-β-lactamase production among extended spectrum β-lactamase (ESBL)-producing Escherichia coli and Klebsiella spp. isolated from clinical specimens. Antibiot (Basel). 2023;13(1):31. 10.3390/antibiotics13010031. 10.3390/antibiotics13010031 PMC10812623 38247589

[35] Hu Y, Matsui Y, Riley LW. Risk factors for fecal carriage of drug-resistant Escherichia coli: a systematic review and meta-analysis. Antimicrob Resist Infect Control. 2020;9:31. 10.1186/s13756-020-0691-3. 32046786 10.1186/s13756-020-0691-3 PMC7014593

[36] Boolchandani M, Blake KS, Tilley DH, Cabada MM, Schwartz DJ, Patel S, et al. Impact of international travel and diarrhea on gut microbiome and resistome dynamics. Nat Commun. 2022;13:7485. 10.1038/s41467-022-34862-w. 36470885 10.1038/s41467-022-34862-w PMC9722912

[37] Mwasinga W, Shawa M, Katemangwe P, Chambaro H, Mpundu P, M’kandawire E, et al. Multidrug-resistant Escherichia coli from raw cow milk in Namwala District, Zambia: public health implications. Antibiot (Basel). 2023;12(9):1421. 10.3390/antibiotics12091421. 10.3390/antibiotics12091421 PMC10525391 37760717

[38] Belayneh SB, Luak CK, Bamboro SA. Pathogenic bacteria in raw milk and milk products in Ethiopia: a decade review of prevalence, contributing factors, and antimicrobial resistance. Syst Rev. 2025;13:20503121251353356. 10.1177/20503121251353356. 10.1177/20503121251353356 PMC12227914 40620332

[39] Liu J, Zhu Y, Jay-Russell M, Lemay DG, Mills DA. Reservoirs of antimicrobial resistance genes in retail raw milk. Microbiome. 2020;8:99. 10.1186/s40168-020-00861-6. 32591006 10.1186/s40168-020-00861-6 PMC7320593

[40] Kumera B, Alemu WM, Adugna A, Teka A, Merid M, et al. Bacteriological quality and public health risk of raw milk in Ethiopia: a systematic review. Discover Appl Sci. 2025. 10.1007/s42452-025-07973-4.

[41] Deneke TT, Bekele A, Moore HL, Mamo T, Almaw G, et al. Milk and meat consumption patterns and the potential risk of zoonotic disease transmission among urban and peri-urban dairy farmers in Ethiopia. BMC Public Health. 2022;22:222. 10.1186/s12889-022-12665-4. 35114957 10.1186/s12889-022-12665-4 PMC8815239

[42] Mpatswenumugabo JP, Mukasafari MA, Ndahetuye JB, Wredle E, Båge R. A systematic literature review of milk consumption and associated bacterial zoonoses in East Africa. J Appl Microbiol. 2023;134:lxad080. 10.1093/jambio/lxad080. 37081784 10.1093/jambio/lxad080

[43] Ahmed MJ, Bhuiyan MI, Chalise R, Mamun M, Bhandari P, et al. One health assessment of farmers’ knowledge, attitudes, and practices (KAPs) on zoonoses in Bangladesh. Sci Rep. 2025;15:1258. 10.1038/s41598-025-85462-9. 39779749 10.1038/s41598-025-85462-9 PMC11711284

[44] Gebremichael D, Tadesse A, Hailemariam F, Hailay B, et al. Investigation of microbial quality of milk and milk products and isolations of some major bacteria in the central and northwestern zones of Tigray, Ethiopia. Vet Med Int. 2024;2024:9989527. 10.1155/vmi/9989527. 39741790 10.1155/vmi/9989527 PMC11685325

[45] Deneke TT, Bekele A, Moore HL, Mamo T, Almaw G, et al. Milk and meat consumption patterns and the potential risk of zoonotic disease transmission among urban and peri-urban dairy farmers in Ethiopia. BMC Public Health. 2022;22:222. 10.1186/s12889-022-12665-4. 35114957 10.1186/s12889-022-12665-4 PMC8815239

[46] Zeru F, Adamu H, Woldearegay YH, Sisay Tessema T, Hansson I, Boqvist S. Occurrence, risk factors and antimicrobial resistance of Campylobacter from poultry and humans in central Ethiopia: A one health approach. PLOS Negl Trop Dis. 2025;19(8):e0012916. 10.1371/journal.pntd.0012916. 40788940 10.1371/journal.pntd.0012916 PMC12360658

[47] Gemeda BA, Wieland B, Alemayehu G, Knight-Jones TJ, Wodajo HD, Tefera M, Kumbe A, Olani A, Abera S, Amenu K. Antimicrobial resistance of Escherichia coli isolates from Livestock and the environment in extensive smallholder Livestock production systems in Ethiopia. Antibiotics. 2023;12(5):941. 10.3390/antibiotics12050941. 37237844 10.3390/antibiotics12050941 PMC10215241

[48] Fujita AW, Werner K, Jacob JT, Tschopp R, Mamo G, Mihret A, Abdissa A, Kempker R, Rebolledo PA. Antimicrobial resistance through the lens of one health in Ethiopia: a review of the literature among humans, animals, and the environment. Int J Infect Dis. 2022;119:120–9. 10.1016/j.ijid.2022.03.041. 35358724 10.1016/j.ijid.2022.03.041 PMC9107604

[49] Ikhimiukor OO, Okeke IN. A snapshot survey of antimicrobial resistance in food-animals in low and middle-income countries. One health;16:100489. 10.1016/j.onehlt.2023.100489 10.1016/j.onehlt.2023.100489 PMC9850425 36683959

[50] Xie Y, Srivastava IM, Jing F, Ma C, Wu X, Chen Y, Du Y, Low XC, Ping Y, Pan J, Gupta A. One health perspective of antibiotic resistance in enterobacterales from Southeast Asia: a systematic review and meta-analysis. Sci Rep. 2025;15:31195.10.1038/s41598-025-31195-8 41413703 10.1038/s41598-025-31195-8 PMC12800116

