# Supplementary material for: Fecal carriage of carbapenemase-producing Escherichia coli and Klebsiella spp. and associated factors among dairy farm milkers in Tigray Region, Northern Ethiopia
Source: BMC Microbiol. 2026 Mar 13;26:389. doi: 10.1186/s12866-026-04943-7 (PMC13097888; doi:10.1186/s12866-026-04943-7)
Supplement: Supplementary file 1 — Additional file 1: Questionnaire [file 12866_2026_4943_MOESM1_ESM.docx]

**Additional files**

**Additional file 1:** Questionnaire

| **Code** | **Checklist or question** | **Response** | **Remark** |
| --- | --- | --- | --- |
| 1. **Socio-demographic characteristics of the interviewee** | | | |
| **101** | **Sex** | Male |  |
|  |  | Female |  |
| **102** | Age (in Years) | 18-34 |  |
|  |  | 35-49 |  |
|  |  | >50 |  |
| **103** | Level of Education | No formal education |  |
|  |  | Primary education |  |
|  |  | Secondary education |  |
|  |  | Diploma and above |  |
| 1. **Questionnaires to assess predictors of fecal carriage of carbapenemase-producing *E. coli* and *Klebsiella* spp. among dairy farm milkers** | | | |
| **201** | **Do you regularly check your health status?** | Yes |  |
|  |  | No |  |
| **202** | **Have you ever undergone any medical instrumentation (e.g., catheterization, endoscopy)?** | Yes |  |
|  |  | No |  |
| **203** | **Have you been admitted to a hospital in the last three months?** | Yes |  |
|  |  | No |  |
| **204** | **Have you ever used antibiotics without a prescription?** | Yes |  |
|  |  | No |  |
| **205** | **Have you had diarrhea in the last three months?** | Yes |  |
|  |  | No |  |
| **206** | Have you had a urinary tract infection in the last three months? | Yes |  |
|  |  | No |  |
| **207** | Have you used any antibiotics in the last three months? | Yes |  |
|  |  | No |  |
| **208** | Do you have a history of any chronic disease? | Yes |  |
|  |  | No |  |
| **209** | Is there a hand-washing facility available on the dairy farm? | Yes |  |
|  |  | No |  |
| **210** | Do you habitually wash your hands before meals and after using the toilet? | Yes |  |
|  |  | No |  |
| **211** | Do you keep your fingernails trimmed? (Observe) | Yes |  |
|  |  | No |  |
| **212** | What is your main source of drinking water? | Tap water |  |
|  |  | Hand-dug well |  |
|  |  | Borehole |  |
|  |  | Bottled water |  |
|  |  | River water |  |
| **213** | Is there a latrine available on the dairy farm? | Yes |  |
|  |  | No |  |
| **214** | Do you have the habit of drinking raw (unpasteurized) milk? | Yes |  |
|  |  | No |  |
| **215** | Do you have the habit of eating raw meat? | Yes |  |
|  |  | No |  |
| **216** | Do you have the habit of eating raw vegetables? | Yes |  |
|  |  | No |  |
| **217** | Is there a disease transmitted through the consumption of milk? | Yes |  |
|  |  | No |  |
